# Wnt Signaling in Stem Cell Maintenance and Differentiation in the *Drosophila* Germarium

**DOI:** 10.3390/genes9030127

**Published:** 2018-02-27

**Authors:** Indrayani Waghmare, Andrea Page-McCaw

**Affiliations:** Department of Cell and Developmental Biology and Program in Developmental Biology, Vanderbilt University School of Medicine, Nashville, TN 37240, USA; indrayani.waghmare@vanderbilt.edu (I.W.); andrea.page-mccaw@vanderbilt.edu (A.P.-M.)

**Keywords:** Wnt, oocyte, follicle cell, escort cell, cap cell, differentiation niche, long-range signaling, stem cell niche

## Abstract

Wnt signaling is a conserved regulator of stem cell behaviors, and the *Drosophila* germarium has been an important model tissue for the study of stem cell maintenance, differentiation, and proliferation. Here we review Wnt signaling in the germarium, which houses two distinct types of ovarian stem cells: the anteriorly located germline stem cells (GSCs), which give rise to oocytes; and the mid-posteriorly located follicle stem cells (FSCs), which give rise to the somatic follicle cells that cover a developing oocyte. The maintenance and proliferation of GSCs and FSCs is regulated by the stem cell niches, whereas differentiation of the germline is regulated by the differentiation niche. Four distinct Wnt ligands are localized in the germarium, and we focus review on how these Wnt ligands and Wnt signaling affects maintenance and differentiation of both germline and follicle stem cells in their respective niches.

## 1. Introduction

Adult stem cells are undifferentiated cells, present in adult tissues, which have the potential to give rise to two or more distinct cell types. They are important for maintaining tissue homeostasis in a wide variety of tissues across the animal kingdom. Some stem cells undergo stereotyped asymmetric divisions, giving rise to a stem-cell daughter and a non-stem cell daughter that differentiates. Other stem cells, however, display more flexible patterns of division and differentiation and can give rise to two stem cells, two differentiated cells, or one of each. The important decision about whether to differentiate or maintain pluripotency is determined by the stem cell niche, a group of cells with molecular signals that maintain the undifferentiated state of the stem cell.

One of the first stem cell niches identified in animals was the germline stem cell niche of the *Drosophila* germarium. Analysis of this niche established that the attachment between niche cells and stem cells was is important for stem cell maintenance but not for niche cell number or function [1,2]. Further, after perturbations inducing loss of stem cells, niche cells promote replenishment of the stem cell population. Thus, early studies of *Drosophila* germline stem cells elucidated three properties of the stem cell niche: (1) The niche defines the physical space within which stem cells can be maintained in an anchorage-dependent manner, (2) stromal cells that form a niche have the ability to rapidly re-program stemness into a cell that enters the niche, and (3) although the niche dictates the stem cell maintenance, the niche itself does not rely on cues from stem cells for survival [1,2]. *Drosophila* models of stem cells continue to provide new discoveries and insights into stem cell biology. This review focuses on how Wnt signaling affects stem cells and their niches during *Drosophila* oogenesis, a process that takes place in an ovarian structure called the germarium.

## 2. Anatomy of the Germarium and an Overview of Egg Chamber Development

**Figure 1:**
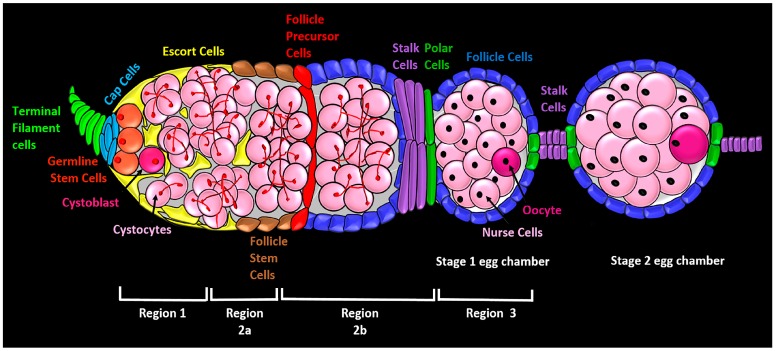
Cell types of the *Drosophila* germarium. The germarium is the anterior-most tissue in the *Drosophila* ovary where oocytes are assembled from the progeny of germline stem cells and follicle stem cells. Assembly proceeds from anterior to posterior (left to right). In a wild-type germarium, terminal filament cells (light green) are found at the anterior end. Cap cells (light blue) and the anterior escort cells (yellow) comprise the germline stem cell niche, providing physical attachments and chemical signals to the germline stem cells (orange). Germline stem cells divide asymmetrically to produce one daughter cell that leaves the stem cell niche and differentiates into a cystoblast (dark pink). The cystoblast enters into the differentiation niche, composed of escort cells (yellow), where it divides four times with incomplete cytokinesis to form a germline cyst composed of 16 cystocytes (pink) joined by cytoplasmic bridges and a cytoskeletal organelle called a fusome (shown as red branching structures in the cystocytes). All these events take place in the anterior-most Region 1. In Region 2a, the oocyte develops further, and at the border between Regions 2a and 2b, the 16-cell cyst passes the follicle stem cells (brown), which give rise to escort cells (yellow), follicle precursor cells (red), polar cells (dark green), stalk cells (purple), and main body follicle cells (dark blue). Follicle cells encapsulate the germline cyst to form a stage 1 egg chamber which buds off the posterior end of the germarium in Region 3. A stage one egg chamber consists of 15 interconnected nurse cells and one oocyte.

Oogenesis in *Drosophila* occurs in the germarium (plural: germaria), which houses two kinds of stem cells: germline stem cells (GSCs) and follicle stem cells (FSCs) (Figure 1). Progeny from these stem cells make up the developing egg, called an egg chamber, and new egg chambers bud off from the posterior of the germarium. At the anterior tip of the germarium within Region 1, cap cells and anterior escort cells form the GSC niche, which promotes GSC maintenance and asymmetric division [1,2,3,4,5,6,7]. (Escort cells are also known as inner germarial sheath (IGS) cells.) Following an asymmetric GSC division, the non-stem cell daughter, called a cystoblast, moves posteriorly to exit the stem cell niche and into a region surrounded by escort cells (Region 1). These escort cells actively promote differentiation of the germline cystoblast, and so this area has been dubbed the differentiation niche [8]. The cystoblast differentiates into cystocyte, which divides four times with incomplete cytokinesis to form a 16-cell germline cyst as it travels posteriorly through Region 1. Lastly, the differentiated germline cyst is encapsulated by follicle cells in Region 2b after it moves through the mid-posterior region (Region 2a) of the germarium. The follicle cells arise from FSCs, and they form the somatic component of the oocyte. Region 2b consists of FSC progeny called follicle precursor cells that divide a few times before giving rise to polar cells, stalk cells and the squamous epithelial main-body follicle cells that surround the developing germline [9,10]. The posterior-most region of the germarium, Region 3, consists of a stage one egg chamber. Thus, the coordinated activities of GSCs and FSCs are critical for formation of normal oocytes [9].

The FSCs have been an area of controversy. Until recently, 2–3 FSCs were thought to reside at the 2a/2b region boundary, just posterior to the escort cells; and these cells gave rise to follicle precursor cells and then to different types of follicle cells, all lying to the posterior of the FSCs [11,12,13,14]. However, a recent study using lineage tracing and live cell imaging argues for the existence of about 14–16 FSCs arranged circumferentially in three layers along the anterior-posterior axis [15]. Further, unlike GSCs, that which are maintained through asymmetric division, FSCs are maintained by ‘population asymmetry’ wherein the fate of the two daughter cells may or may not be identical. This results in stochastic amplification or loss of individual FSCs [15]. Lineage tracing shows that FSCs in different layers give rise to different cell types: the anteriorly located FSCs give rise to escort cells that lie anterior to the FSCs, whereas the posteriorly located FSCs give rise to the traditionally described follicle precursor cells that differentiate into polar cells, stalk cells and the squamous epithelial main body follicle cells. Because FSCs can move between layers, they give rise to multiple cell types and are multipotent [15]. An interesting recent study also shows that the fate of follicle precursor progenies is not stereotyped, and in fact is determined by multiple signaling pathways [16]. These new findings represent important revisions to our understanding of follicle stem cells.

In a wild type germarium from a well-fed female, many stages of oogenesis are evident at one time. A GSC divides on average once a day and is maintained in the niche with a half-life of four to five weeks. In addition to GSCs and cystoblasts, two-, four-, eight- or 16-cell cysts can be seen in a germarium [5,12]. The GSCs, cystoblasts, and cysts can be identified on the basis of the morphology of distinct cytoskeletal structures called spectrosomes and fusomes. The GSCs and cystoblasts both contain round spectrosomes, but the cell types can be distinguished by the placement of the spectrosomes: within each GSC, the spectrosome abuts the cap cells of the niche, whereas within each cystoblast, the spectrosome is located centrally inside the cell [17]. In contrast to the GSCs and cystoblasts, the cysts contain fusomes that extend through the ring canals connecting the cystocytes of each cyst, displaying a branched morphology that is distinct from the round spectrosome [18]. 

Wnt signaling has important functions in maintaining stem cells and in promoting differentiation of stem cell progeny, as we will describe in the next sections. Wnt signaling is also important, however, in the development of the germarium architecture during pupal metamorphosis. Fortunately, the sophisticated conditional genetics available in *Drosophila* can tease apart the role of Wnt signaling in the homeostatic process of oogenesis vs. Wnt signaling during germarium development.

## 3. The Wnt Signaling Pathway 

**Figure 2:**
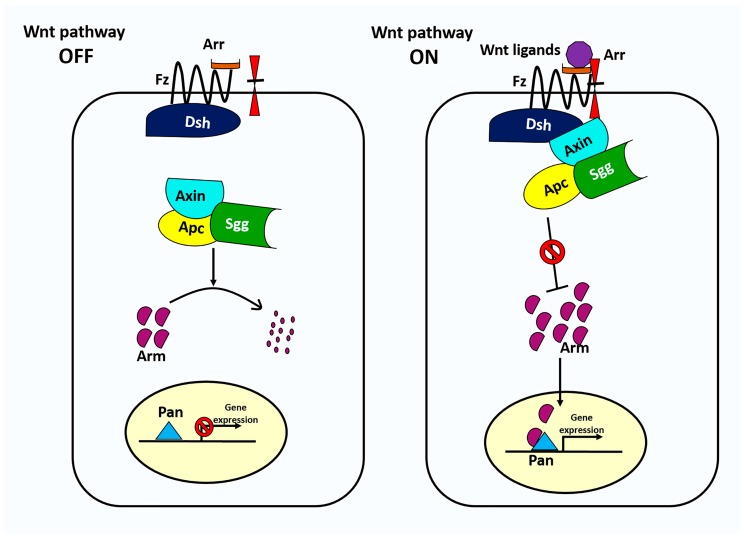
The canonical Wnt signaling pathway in *Drosophila*. In the absence of Wnt signaling (left), the destruction complex phosphorylates the transcriptional regulator Arm, targeting it for subsequent destruction by the proteasome. The destruction complex is a multiprotein complex that includes APC, Axn, and Sgg. In the presence of Wnt ligand (right), Wnt binds to Fz and Arr, triggering translocation of the destruction complex to the inner plasma membrane via Dsh and its subsequent inactivation. Without phosphorylation, the concentration of Arm rises and enters the nucleus where it binds to nuclear transcription factor Pan to regulate Wnt target genes.

The Wnt signaling pathway (Figure 2) is an evolutionarily conserved pathway that participates in several biological processes such as embryonic development, cell division, cell survival [19], stem cell maintenance [20], cell adhesion and migration [21], and planar cell polarity (PCP) [22,23]. Wnt signaling has been heavily studied with respect to stem cells [24,25,26,27]. The Wnt extracellular ligand was originally identified in two systems, as a patterning gene in *Drosophila*, *wingless* (*wg*) [28], and as a proto-oncogene in mammary tumors, *Int-1* [29,30]. The merger of these two nomenclatures resulted in the name Wnt [31]. The steps of Wnt pathway signaling are highly conserved, and we review them here primarily to introduce the *Drosophila* names for the conserved components (Table 1). Canonical signaling is initiated when the Wnt ligand binds to the Frizzled (Fz) receptor and Arrow (Arr) co-receptor. Activated Wnt signaling results in the accumulation of the key downstream effector, Armadillo (Arm), a transcriptional regulator which enters the nucleus and alters gene expression. When Wnt signaling is off, Arm protein is phosphorylated and destroyed by the destruction complex, which consists of Adenomatous polyposis coli (APC), Axin (Axn), and Shaggy (Sgg) kinase. When Wnt signaling is on, the destruction complex is translocated to inner plasma membrane via Dishevelled (Dsh), and no longer promotes destruction of Arm. Once Arm accumulates, it enters the nucleus and partners with transcription factors such as Pangolin (Pan) and Pygopus (Pygo) to regulate the transcription of target genes (reviewed in [21,32,33]).

**Figure 3:**
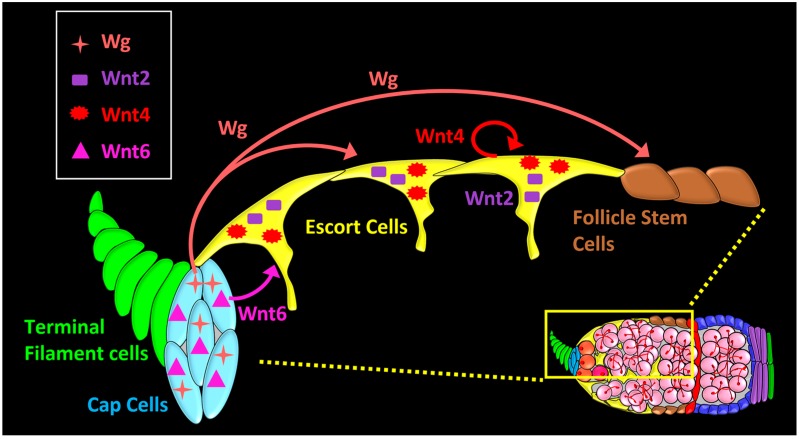
Localization, source, and signaling range of Wnt ligands in the *Drosophila* germarium. *wg* and *Wnt6* ligands are expressed in cap cells. Wg acts on escort cells (short-range), and on follicle stem cells (FSCs) (long-range). Wnt6 from cap cells is required for survival of anterior escort cells, and maintenance of germline stem cells (GSC) niche. *Wnt2* and *Wnt4* are expressed in the escort cells for the maintenance of the differentiation niche.

The *Drosophila* genome has seven Wnt family genes, compared to 19 in human and mouse [34]. These *Drosophila* genes are called *wg* (aka *Wnt1*), *Wnt2*, *Wnt4*, *Wnt5*, *Wnt6*, *WntD* (aka *Wnt8*), and *Wnt10*. Five of the Wnt genes have been reported to be expressed in the fly germarium [35,36,37,38]. Four of these Wnts can be detected by RNA in situ hybridization, RNA-Sequencing (RNA-Seq), or immunohistochemistry: *wg* and *Wnt6* are highly expressed in cap cells [35,37,38,39], and *Wnt2* and *Wnt4* are expressed in both cap cells and escort cells (Figure 3) [35,36]. In contrast, *Wnt5* is expressed at low levels and has been detected in escort cells only after dissociation and cell-sorting [36]. As secreted proteins, Wnts can initiate signaling in cells that are located several cells away from the source cells [40,41,42,43,44], and there is evidence for long-range Wg signaling in the germarium [37,38,39,45]. Thus, there are important questions about specificity: how much does one Wnt protein relay specific information, distinct from other Wnts, and how is signaling spatially targeted to activate only the appropriate cells?

## 4. Wnt Pathway Functions in the Germarium

Wnt signaling is important in all aspects of stem cell behavior in the germarium. We use the term Wnt signaling because it is sometimes not clear which Wnt ligand is responsible for signaling, as the downstream signaling pathway is shared by the Wnt ligands. Below we discuss how Wnt signaling affects the maintenance of GSCs, FSC differentiation and proliferation, and pupal development of the germarium. Finally, we address what is known about how long-distance Wnt signaling is regulated in the germarium.

### 4.1. How Wnt Signaling Affects Maintenance of Germline Stem Cells

The GSCs are maintained by their niche. The first niche cells recognized as critical to GSC maintenance were the cap cells. Cap cells contribute to the GSC niche by two distinct mechanisms: (1) they produce Decapentaplegic (Dpp) ligand to induce paracrine Dpp signaling in GSC, which is essential for maintaining stemness [5]; and (2) they are involved in anchoring GSCs to the niche via expression of E-cadherin [2]. A new report establishes that the GSC niche contains a second cell type, anterior escort cells, which are also absolutely required to maintain GSCs [46]. Like cap cells, anterior escort cells maintain GSCs by expressing Dpp to promote signaling [1,46,47,48,49], and when *dpp* is knocked down in escort cells, GSCs are lost [48]. Also like cap cells, anterior escort cells physically attach to GSCs through E-cadherin, and when E-cadherin is knocked down in escort cells, GSCs are lost from the niche [46]. 

When escort cells are ablated or their function is hindered, there is a pronounced effect on germline differentiation (discussed in the next section) and any remaining escort cells tend to cluster toward the anterior [46]. When these persistent anterior-most escort cells are lost, GSCs are lost from the niche. GSCs are also lost when escort cells are directly induced to apoptose or when they are deprived of Wnt signaling [46]. These results indicate that anterior escort cells are a critical GSC niche cell-type. Interestingly, anterior escort cells are maintained by Wnt6 emanating from the cap cells, and when Wnt6 is knocked down in cap cells in adults, anterior escort cells and GSCs are lost [46]. Thus Wnt6, emanating from cap cells, may be a signal that promotes survival of anterior escort cells to coordinate the two niche cell types. 

A relatively recent study proposed that the GSC niche boundary is defined by cap cell derived Wnt ligands, Wg and Wnt6 [35]. These Wnts act redundantly to inhibit Dpp signaling outside of the GSC niche by upregulating the expression of Dpp receptor *thickveins* (*tkv*) in escort cells. Interestingly, Tkv expression in escort cells does not participate in signal transduction but instead acts as ‘receptor sink’ to prevent ectopic activation of Dpp signaling in the differentiating germline. At the transcriptional level, chromatin immunoprecipitation and luciferase reporter assays reveal that the Wnt effector Arm occupies a regulatory region of *tkv* to drive its expression in escort cells [35]. The GSC niche is thus defined by its cell types, the signals it produces, and the extent to which these signals spread, all of which together determine the position of GSCs.

### 4.2. How Wnt Signaling Affects GSC Differentiation

GSC daughters exit the niche and undergo differentiation in response to several cues that are provided by the differentiation niche. The differentiation niche consists of escort cells that surround the developing cystoblasts and cystocytes [3,4,8], and it promotes differentiation via intercellular communication and physical contact between escort cells and the developing germline [50,51]. Thus, the survival and integrity of escort cells is crucial for proper differentiation, and mutations that disrupt escort-germ cell communication result in a failure of differentiation [8,35,36,46,47,50,51,52,53,54,55,56,57,58,59]. The fact that a differentiation niche is required indicates that stem cells do not inherently differentiate, and further suggests that the role of the stem cell niche may be to control stem cell proliferation and placement, rather than to inhibit their differentiation.

Germline differentiation phenotypes are easily recognized by the large increase in spectrosome-containing undifferentiated germline cells. Typically, a wild type germarium contains 2–3 spectrosome-containing cells, two GSCs, and one cystoblast [18]. *Wnt4* is expressed in escort cells, and several studies have shown that loss of *Wnt4* from escort cells causes differentiation defects resulting in germaria that contain more than 3 spectrosome containing cells [35,36,46,50,51,52]. This phenotype has been called an ovarian tumor. The cystoblasts that fail to undergo differentiation are in limbo, as they are neither stem cells nor fully differentiated. 

Wnt4 signaling is autocrine within escort cells. Escort-cell loss of positive regulators of Wnt signaling, including *fz*, *fz2*, *dsh*, *arr*, or *arm* gives rise to ovarian tumors [8,35,36,50,51,52]. Conversely, escort-cell overexpression of constitutively active arm (*arm^S10^*) rescues the ovarian tumor phenotype [51]. Loss of *Wnt4* expression or Wnt activity in escort cells also leads to expansion of GSC niche wherein escort cells ectopically express *dpp* transcripts and promote weak ectopic Dpp signaling in some spectrosome-containing germline cells. As expected, escort-cell knockdown of *dpp* rescues the *Wnt4* loss of function phenotype, suggesting that Wnt4 signaling inhibits *dpp* expression in escort cells [51]. Reciprocally, downregulation of *dpp* signaling in all escort cells induces ectopic Wnt signaling in anterior escort cells, suggesting that Wnt and Dpp pathways mutually antagonize each other in escort cells to delineate GSC niche from differentiation niche [51]. Similarly, another study finds that loss of *dpp* rescues the ovarian tumor phenotype caused by escort-cell knockdown of *dsh* [36]. Thus, Wnt signaling is required for restricting the extent of GSC niche and promoting GSC differentiation.

Another mechanism by which escort-cell Wnt4 ensures proper germline differentiation is by responding to transposon activity, which causes disruption in genomic integrity [50]. Genomic integrity is maintained in part by the piwi interacting RNA (piRNA) pathway, mediated by protein-RNA complexes that target and silence transposable elements at transcriptional and translational levels downstream of the histone methyltransferase, Eggless [58,60,61,62]. In escort cells, increasing transposon activity by knocking down *eggless*, *piwi* or *flamenco* results in both a reduction of *Wnt4* expression and an accompanying ovarian tumor phenotype [50,57]. Wnt4 promotes differentiation by promoting escort cell encapsulation of differentiating cystocytes, via gap junctions and adherens junctions. The loss of gap-junction and adherens junction components Innexin, E-cadherin, and Arm also cause a failure of differentiation [50,56,63]. Thus, Wnt4 acts to safeguard genomic integrity through its escort-cell autonomous role in promoting germline differentiation.

In addition to Wnt4’s specific roles in restricting the GSC niche and promoting physical contact between differentiating germline and the escort cells, Wnt2 and Wnt4 act redundantly to maintain the differentiation niche by promoting proliferation and survival of escort cells [36]. Hyperactivation of the Wnt signaling pathway by overexpression of constitutively active *arm^S10^* results in increased number of escort cells, from 30–35 in a normal germarium to about 130 in mutant germarium [36,46]. Interestingly, this hyperactivation of Wnt signaling in escort cells does not affect germline differentiation [36]. 

Wnt2- and Wnt4-mediated Wnt signaling also promote escort-cell expression of genes that eliminate reactive oxygen species (ROS) such as *Glutathione S-transferases* (*GstD2*, *GstD4*, *GstD10*, and *GstE3*) [36]. GSTs and Catalases remove hydrogen peroxide from cells [64,65]. Importantly, overexpression of *GstD2* and *Catalase* rescues Wnt4 differentiation defects in escort cells [36]. Thus, Wnt signaling maintains a reduced redox state, and this cellular environment is critical to promote differentiation. Whether there is interplay between the redox environment and transposons in escort cells has not been explored.

### 4.3. How Wnt Signaling Affects Follicle Stem Cells Differentiation and Proliferation

While the GSC niche is located in close proximity to the GSCs, the FSC niche is complex. This niche consists of escort cells adjacent to the FSCs, the basement membrane underlying them [66], and signals emanating from distant cells, including Wg from the cap cells [37,38,39,67]. *wg* expression in the germarium was first reported in cap cells [37], but its expression pattern remains perplexing. Although by antibody staining, Wg protein is observed in all 5–7 cap cells [38,39], *wg* message is much more sporadic, with only 1–3 cap cells expressing a *wg-lacZ* enhancer trap, and about 20–30% of germaria displaying no expression of the enhancer trap at all [37]. Our lab has obtained similar results [68] using a different *wg-Gal4* enhancer trap, expected to faithfully recapitulate *wg* expression because Gal4 is inserted in the endogenous *wg* locus [69]. These results raise the possibility that Wg protein levels are not contributed evenly by cap cells but are subject to unknown regulatory mechanisms. Unlike in the embryonic ectoderm, *wg* expression in cap cells is independent of Engrailed (En) or Hedgehog (Hh) signaling [37]. Regardless of how Wg levels are regulated, the most obvious function of Wg in the cap cells is to regulate distant FSCs.

Since the first report on Wg/Wnt in the germarium [37], several studies have investigated the role of Wnt signaling in FSC regulation [15,16,35,38,39,45] and concluded that cap-cell produced Wg promotes FSC survival and proliferation [37,38,39]. Overexpression throughout the germarium of *wg*, *dsh*, *fz* or *arm^S10^*, all positive regulators of Wnt signaling, results in overproduction of somatic stalk cells that connect the developing egg chambers [38,39]. The stalk cells derive from FSCs and an increase in stalk cell number is caused by overproliferation of FSCs and not ectopic mitoses in stalk cells [39]. In contrast, loss of *wg*, either from a *wg* temperature-sensitive allele or *wg*-*RNAi*, results in fused egg chambers, a result of insufficient follicle cells, indicating that Wg signaling is required for FSC proliferation [38,39]. It is important to note that the FSC overproliferation phenotype caused by overexpression of *wg* is weaker than the phenotype caused by increases in Hh or Notch signaling activities, suggesting that multiple pathways interact to regulate FSCs [45]. 

It has been noted that FSCs are lost when Wnt signaling is either upregulated or downregulated [39]. Recent work sheds light on this mechanism. As mentioned previously, lineage-tracing studies recently discovered that there are more follicle stem cells and that they give rise to more cell types than previously recognized [15]. Although these FSCs are organized circumferentially into three rings, or layers, along the anterior-posterior axis, their positions are not fixed and they can move within the three layers. Typically, fewer FSCs reside in the anterior layers than in the posterior layer [15]. Further, the anterior FSCs give rise to escort cells at a rate 4 times slower than the posterior FSCs give rise to follicle cells. These observations suggest that spatial information regulates their proliferation and cell fate choice [15]. Interestingly, FSC placement in the niche and their lineage choices are strongly regulated by Wnt signaling. Loss of Wnt signaling in FSCs results in a specific loss of FSCs from the anterior layer that supplies escort cells, an increase in FSCs in the posterior layer, and an increase in follicle cells; thus, without Wnt signaling FSCs moved posteriorly and differentiated into follicle cells without self-renewal. Reciprocally, increased Wnt pathway activity in FSCs promoted the exit of FSCs from the posterior layer to the anterior layer, thereby causing loss of follicle cell production and an increase in number of escort cells. Thus, Wnt signaling determines the fate of FSC progeny between escort cell and follicle cell [15]. A recent study found that Wnt signaling also determined which of three follicle cell fates would be adopted by follicle precursor cells, with high Wnt signaling resulting in more stalk and polar cells at the expense of main body cells [16].

There is a surprising discrepancy between reports that the Wg signal, emanating from cap cells, promotes an increase in follicle cell numbers [38,39], and the recent report that increased Wnt pathway activity in FSCs decreases follicle cell fates [15]. What may account for the difference in follicle cell numbers in these types of studies? Importantly, increases in follicle cell numbers occur when the Wg ligand is expressed from outside the FSCs themselves, whereas fewer follicle cell numbers occur when the Wnt pathway, downstream of all Wnt ligands, is altered in the FSCs themselves. One possibility is that there is some ligand specificity that makes *wg* expression distinct from Wnt pathway activation. Another possibility is that these genetic manipulations may activate Wnt signaling to differing extents within the FSCs. Yet a third possibility is that *wg* expression promotes FSC proliferation in an indirect manner, via an unknown intermediate. Further studies will be needed to resolve these apparently contradictory results.

Distinct from the germline tumor phenotype resulting from defects in the differentiation niche, somatic follicular tumors result from simultaneous mutations in *Posterior sex combs* (*Psc*) and *Suppressor of zeste two* (*Su(z)2*) [70]. These tumor masses are ectopically formed in Regions posterior to FSC location and derive from mutant FSCs that are basally extruded from the epithelium without disrupting the basement membrane. The tumorous growth of mutant FSC relies on canonical Wg signaling as overexpression of *dominant negative TCF* (*TCF^DN^*) or *wg-RNAi* in mutant clones rescues the tumor phenotype. Further, independent of the canonical Wg signaling pathway, the extrusion of mutant FSCs relies on the non-canonical Wnt-PCP pathway [70].

### 4.4. How Wnt Signaling Affects Pupal Development of the Germarium

As described above, Wnt signaling is important for nearly all aspects of stem cell biology in the germarium, as progeny of the follicle and germline stem cells interact to produce new egg chambers in a continuous manner. However, Wnt signaling is also important for establishing the architecture and functioning of the germarium [70]. Ovarian morphogenesis begins during late third-instar and continues through pupal stages. The larval ovary is divided into individual ovarioles by the migration of the apical cells between the terminal filament cells [9]. *Wnt4* is expressed early on in all apical cells during ovarian morphogenesis and continues to express in apical cells as they migrate basally to delineate the basal cell population, and loss of *Wnt4* in these cells disrupts their migration by disrupting focal adhesions [70]. The resulting ovarioles have a defective ovariolar sheath, leading to morphological abnormalities that appear as collapsed ovarioles [71]. Wnt6 is also important for some aspect of ovarian morphogenesis, because the loss of Wnt6 throughout development, either specifically in cap cells or throughout the whole animal, results in a germline tumor [35,46], whereas the loss of *Wnt6* only in adults results in the loss of GSCs but not germline tumors [46].

## 5. Long-Range and Short-Range Wnt Signaling

Wnt ligands can activate cell signaling in neighboring cells in a juxtracrine manner, and also at long range in distant cells 50–100 µm away. Because Wnt proteins are secreted [44], it has long been thought that they travel extracellularly from source cells to target cells to act as morphogens and signaling molecules. Several different models have been proposed to explain Wg spreading in extracellular environment: extracellular diffusion of Wnt ligands mediated by heparan sulfate proteoglycans (HSPGs), formation of stable extracellular complexes with carrier proteins, packaging into small vesicles called exosomes, and formation of filopodia-like structures called as cytonemes [72]. Recently, however, challenges have arisen to the idea that Wnt ligands act at long distance from their source [34,69,73]. Because the best model of Wnt spreading has been the *Drosophila* wing disc, the most serious challenge came from a study that eliminated Wg spreading in the wing disc by tethering endogenous Wg to the cell membrane, so that it could signal only in an autocrine or juxtracrine manner [69]. These flies homozygous for tethered Wg, without any wild-type free Wg protein, could survive to adulthood and displayed normal wing patterning, indicating that Wg spreading is not necessary for wing patterning. However, observations that these tethered-Wg flies have poor viability and fertility suggest that Wg extracellular spreading may be required for other aspects of development, and we discuss the role of HSPGs in spreading Wg in the germarium below.

The tethered-Wg fly results also do not address the possibility that Wnt ligands travel long distances on cytonemes. Cytonemes are long thin cytoplasmic protrusions extending across many cell diameters to mediate cell-cell signaling between cells that are otherwise not adjacent [74,75]. Cytonemes promote juxtacrine signaling by bringing ligands and receptors in close proximity to activate signaling in recipient cells. Cytoneme-delivery of Wnt ligands or receptors has been reported in the *Drosophila* wing disc [76], embryonic development of zebrafish neural plate [77], and chick dermomyotome [78]. In the *Drosophila* ovary however, cytonemes have been described only in the context of Hh signaling where cap cells send out cytonemes to activate Hh signaling in escort cells to regulate GSC niche [48].

HSPG-dependent Wg spreading has been identified in the germarium. This mechanism for Wg spreading was first identified in the wing disc [79] where the HSPG Dlp (Dally like protein) is expressed in a pattern complementary to that of Wg [80,81,82]. Dlp acts as an exchange factor with the Fz receptor to promote the long-range spread of Wg and simultaneously inhibit short-range Wg activity. The ability of Dlp to act as a positive or negative regulator of Wg activation is determined by the relative ratios of cell-surface Dlp, Fz and Wg [81]. In contrast, Dally, which is another HSPG in flies, only positively regulates the spread of extracellular Wg [80]. The core proteins of Dlp and Dally are required for ligand binding, whereas other domains and the polysaccharide glycosaminoglycans (GAG) chains might have an auxiliary function [81]. In the germarium, Dlp-mediated Wg spreading appears to function similarly to the wing disc. Like in the wing disc, Dlp is observed in a pattern complementary to that of Wg, with Dlp localized to the terminal filament cells and escort cells [38,83], whereas Wg is localized to cap cells. Knockdown of *dlp* in somatic cells results in decreased extracellular Wg spreading from cap cells and a reduction of long-range Wg signaling activity in the germarium [38]. Knockdown of *dlp* also phenocopies the *wg* loss of function phenotype, causing a reduction in the number of stalk cells, reflecting decreased proliferation of FSCs [38,39]. Thus, Dlp promotes long-range spread of Wg from cap cells to FSCs.

In the germarium Dlp is negatively regulated by the matrix metalloproteinase Mmp2. Loss of *Mmp2* phenocopies the overexpression of *wg*, which can be suppressed by reducing the level of *wg*; and overexpression of *Mmp2* phenocopies the loss of function of either *dlp* or *wg*, which can be suppressed by overexpressing *wg* [38]. Thus, Mmp2 inhibits Dlp-mediated long range Wg signaling in FSCs. In addition, in S2R+ cells, Dlp is a substrate for Mmp2 proteolytic cleavage [38]. Thus, Wg produced and secreted by cap cells spreads in the extracellular space by binding to Dlp to activate Wg signaling in FSCs, and Mmp2 mediated cleavage of Dlp provides a ‘brake’ to prevent excess Wg signaling in FSCs. Interestingly, Dlp is also required for cytoneme-mediated signaling in the wing disc [84], raising the possibility that these two signaling mechanisms may be intertwined in the germarium.

## 6. Conclusions and Perspectives

Wnt signaling is critical for stem cell behavior in the *Drosophila* germarium, as collectively Wnt signaling controls stem cell maintenance, proliferation, fate determination, and survival of somatic cells important for maintaining stem cell functions. Four different Wnt ligands function in the germarium—Wg, Wnt2, Wnt4, and Wnt6—and with the exception of Wnt2, each appears to act non-redundantly. It is still not clear what provides the specificity for each of these Wnt signals—is it the molecular identity of the Wnt ligand, or the spatial positioning of the signal-emitting cell with respect to target cells, or the level of Wnt protein? Questions remain about the extracellular spreading of Wnt ligands—for example, can the tethered Wg construct support oogenesis? If four distinct Wnt ligands are spreading from different sources in close proximity, how does each ligand target only the appropriate receiving cells? Does Dlp mediate the spreading of multiple Wnt ligands? *Drosophila* has outstanding genetic tools, such as the highly flexible Gal4/UAS system, which allows gene activation or inactivation in each germarium cell type independently, affording the ability to alter signal-sending cells or signal-receiving cells. When used with the temperature-sensitive Gal80 inhibitor, such manipulations can be performed with temporal as well as spatial specificity. Thus, future research is likely to be able to address these questions.

## Figures and Tables

**Table 1:** *Drosophila* genes encoding the main components of canonical Wg/Wnt signaling pathway.

Fly Gene Name	Vertebrate Family	Function
*wingless* (*wg*)/*Wnt1* *Wnt2* *Wnt4* *Wnt5* *Wnt6* *Wnt8/WntD* *Wnt10*	Wnt	Ligand (Positive regulator)
*frizzled* (*fz*) *frizzled 2 (fz2)* *frizzled3 (fz3)* *frizzled 4* (*fz4*)	Frizzled (Fz)	Receptor (Positive regulator)
*arrow* (*arr*)	Low-density lipoprotein receptor-related protein 5/6 (LRP5/6)	Co-receptor (Positive regulator)
*dishevelled* (*dsh*)	Dishevelled (Dsh or Dvl)	Signal transduction (Positive regulator)
*APC-like* (*Apc*) *Adenomatous polyposis coli 2* (*Apc2*)	Adenomatous Polyposis Coli (APC)	Signal transduction (Negative regulator—component of the ‘destruction complex’)
*Axin* (*Axn)*	Axin (Axn)	Signal transduction (Negative regulator—component of the ‘destruction complex’)
*shaggy* (*sgg)*	Glycogen Synthase Kinase 3β (GSK3β)	Signal transduction (Negative regulator—component of the ‘destruction complex’)
*armadillo* (*arm*)	β-Catenin (β-Cat)	Transcriptional co-activator (Positive regulator)/cell-adhesion molecule
*pangolin* (*pan*)	T-cell factor ( TCF)	Transcriptional co-activator (Positive regulator)
